# Nontrivial coupling of light into a defect: the interplay of nonlinearity and topology

**DOI:** 10.1038/s41377-020-00371-y

**Published:** 2020-08-19

**Authors:** Shiqi Xia, Dario Jukić, Nan Wang, Daria Smirnova, Lev Smirnov, Liqin Tang, Daohong Song, Alexander Szameit, Daniel Leykam, Jingjun Xu, Zhigang Chen, Hrvoje Buljan

**Affiliations:** 1 The MOE Key Laboratory of Weak-Light Nonlinear Photonics, TEDA Applied Physics Institute and School of Physics, Nankai University, Tianjin, 300457 China; 2 Faculty of Civil Engineering, University of Zagreb, A. Kačića Miošića 26, 10000 Zagreb, Croatia; 3 Nonlinear Physics Centre, Research School of Physics, Australian National University, Canberra, ACT 2601 Australia; 4 Institute of Applied Physics, Russian Academy of Science, Nizhny Novgorod, 603950 Russia; 5 Collaborative Innovation Center of Extreme Optics, Shanxi University, Taiyuan, 030006 Shanxi PR China; 6 Institut für Physik, Universität Rostock, Albert-Einstein-Strasse 23, 18059 Rostock, Germany; 7 Center for Theoretical Physics of Complex Systems, Institute for Basic Science (IBS), Daejeon, 34126 Republic of Korea; 8 Basic Science Program, Korea University of Science and Technology, Daejeon, 34113 Republic of Korea; 9 Department of Physics and Astronomy, San Francisco State University, San Francisco, CA 94132 USA; 10 Department of Physics, Faculty of Science, University of Zagreb, Bijenička c. 32, 10000 Zagreb, Croatia

**Keywords:** Nonlinear optics, Photonic devices

## Abstract

The flourishing of topological photonics in the last decade was achieved mainly due to developments in linear topological photonic structures. However, when nonlinearity is introduced, many intriguing questions arise. For example, are there universal fingerprints of the underlying topology when modes are coupled by nonlinearity, and what can happen to topological invariants during nonlinear propagation? To explore these questions, we experimentally demonstrate nonlinearity-induced coupling of light into topologically protected edge states using a photonic platform and develop a general theoretical framework for interpreting the mode-coupling dynamics in nonlinear topological systems. Performed on laser-written photonic Su-Schrieffer-Heeger lattices, our experiments show the nonlinear coupling of light into a nontrivial edge or interface defect channel that is otherwise not permissible due to topological protection. Our theory explains all the observations well. Furthermore, we introduce the concepts of inherited and emergent nonlinear topological phenomena as well as a protocol capable of revealing the interplay of nonlinearity and topology. These concepts are applicable to other nonlinear topological systems, both in higher dimensions and beyond our photonic platform.

## Topological photonics: nonlinear coupling

An engineered photonic system has provided insights into the interplay between nonlinearity and topology. Shiqi Xia and coworkers used laser-writing technique to fabricate refractive-index patterns that form a Su-Schrieffer-Heeger lattice in a nonlinear optical crystal of strontium-barium-niobate (SBN:61), a well-known photorefractive material. Due to the presence of the nonlinearity, two optical beams of sufficient power propagating in different directions can, when they collide, couple into a non-trivial interface state in the system, which is otherwise not accessible due to topological protection. Theoretical analysis correctly explains the observed behavior and the nonlinear coupling phenomenon. The researchers say that their findings are of a general nature and can be applied to other nonlinear topological systems.

## Introduction

Topological photonics has become one of the most active research frontiers in optics over the last decade^1,2^. The initial ideas were drawn from condensed matter physics, where the concept of topology was found to be crucial for understanding the celebrated quantum Hall effect (QHE)^3,4^ and, later on, for the development of topological insulators^5–7^. In 2008, Raghu and Haldane proposed that the Bloch bands of photonic crystals designed with time-reversal symmetry-breaking elements could have nontrivial topological invariants^8,9^, namely, the non-zero Chern numbers. When two materials with different topological invariants are interfaced, bulk-edge correspondence^2,10,11^ guarantees the existence of topological edge states, which enjoy robust unidirectional propagation. Such correspondence holds in both quantum and classical wave systems, which inspired the first observation of the unidirectional propagation of electromagnetic waves in the microwave regime^12^. Topological states of light and related phenomena were later realized in various systems, including photonic lattices^13^, ring resonators^14^, and metamaterials^15^ (see Ref. ^2^ for a recent review).

In electronic systems, the interplay of topology and quantum many-body interactions can result in intriguing topological states of matter such as the fractional QHE^4,16^. An analogous yet distinct avenue of research addresses the interplay of topology and nonlinearity in photonics. In conventional linear systems, the amount of energy present in each eigenmode remains constant during time evolution. When nonlinearity is introduced, however, it shuffles the energy between the eigenmodes, which brings back memory of the pioneering numerical experiment by Fermi et al., who studied the thermalization induced by nonlinear coupling in 1955^17^. Their discovery of the recurrence to a state very close to the initial condition in a surprisingly short time is rooted in the underlying integrability of the system. Such a recurrence was recently observed with nonlinear optical spatial waves^18^. It is natural to wonder whether the eigenmodes of a topological system can be coupled by nonlinearity and, if so, how the nontrivial topology can be reflected in the subsequent dynamics, especially in the coupling of topologically protected edge states.

Thus far, nonlinear topological effects have been investigated far less than their linear counterparts, although nonlinearity inherently exists in many topological photonic platforms, such as waveguide arrays, coupled resonators, and metamaterials^19–31^. Seeking unique functionalities and device applications, research in nonlinear topological photonics has been focused mainly on edge solitons in topological structures^21,23,32–34^, nonlinearity-induced topological transitions^24,25^, nonlinear frequency generation^35–37^, and topological lasing^38–40^. Despite these efforts, the fundamental issue of the nonlinear coupling of eigenmodes in topological systems remains largely unexplored.

Here, we demonstrate nonlinearity-induced coupling of light into topologically protected edge states using a photonic platform and develop a general theoretical framework for interpreting the mode-coupling dynamics in nonlinear topological systems. The experimental results are obtained in photonic Su-Schrieffer-Heeger (SSH) lattices^41^ fabricated with a laser-writing technique in a nonlinear crystal. We observe that only under nonlinear excitation can a light beam traveling from the bulk to the edge of a nontrivial SSH lattice be coupled to a topologically protected edge state. Furthermore, the nonlinear interaction of two beams at opposite incident angles is also observed, coupling into a topological interface state that depends strongly on their relative phase. Our theory explains these observations well: under proper nonlinear excitation, the profile of the beam propagating along the edge (or interface) waveguide is inherited from that of the underlying linear topological system, overlapping more than 98% with the linear topological edge states and with propagation constants residing in the band gap. When the nonlinearity is stronger than a certain critical value, however, the nonlinear eigenvalue of the edge state moves out of the gap and emerges above the first band, indicating that the localization is now dominated by nonlinearity. The concepts introduced in this paper are generally applicable to nonlinear topological systems.

The SSH lattice exhibits two topologically distinct (Zak) phases, representing a prototypical one-dimensional (1D) topological system with chiral symmetry^2,41,42^. SSH models have been implemented in a variety of platforms, including photonics and nanophotonics^43–49^, plasmonics^50,51^, and quantum optics^52–55^, and particularly in the context of topological lasing^38,56–58^. Such SSH-type models with driven nonlinearity have also attracted great attention^19,24,30,32,34–36,59^. In particular, nonlinearity has been employed for spectral tuning^30^ and time-domain pumping^59^ of topological edge states and for the generation of topological gap solitons^32,34^ in such systems.

## Results

**Fig. 1:**
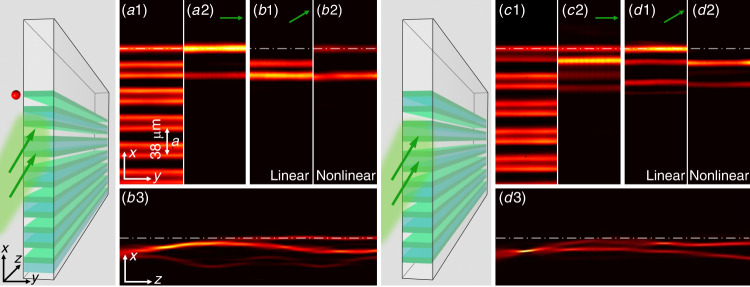
Comparison of the edge excitation between topologically trivial and nontrivial SSH lattices. The two illustrations show the tilted excitation (green arrows) for nontrivial (left) and trivial (right) lattices, where the red dot marks the position of the nontrivial edge. (a1–b2) The experimental results obtained with the nontrivial lattice, where (a1) shows the written SSH waveguide lattice examined by a probe beam, (a2) shows the output of a topological edge state under normal (straight) excitation, and (b1) and (b2) show the outputs with a tilted beam (*k*_x_ = −1.4*π*/*a*) in linear and nonlinear cases. (b3) The simulation results show a side-view (up to a crystal length of 20 mm) of the beam dynamics under nonlinear excitation. The right panels (c1–d3) have the same layout as the left panels except that the results are obtained with the trivial lattice. The white dashed-dotted line marks the edge position of the SSH lattice in all figures

We study the propagation of light in photonic lattices with a refractive-index variation given by $n_0 + \delta n_L\left( {\mathbf{x}} \right) + \delta n_{NL}\left( {\left| \psi \right|^2} \right)$, where *n*_0_ is the constant part of the material’s index of refraction, *δn*_*L*_(**x**) describes the linear photonic lattice, which is uniform along the propagation axis *z*, and $\delta n_{NL}\left( {\left| \psi \right|^2} \right)$ is the nonlinear index change, which depends on the intensity of the light (with *ψ*(**x**, *z*) being the complex amplitude of the electric field). In the paraxial approximation, the propagation of the light is modeled by the following Schrödinger-type equation with a nonlinear term:

1 $$i\frac{{\partial \psi }}{{\partial z}} = - \frac{1}{{2k_0}}\nabla ^2\psi - \frac{{k_0\delta n_L\left( {\mathbf{x}} \right)}}{{n_0}}\psi - \frac{{k_0\delta n_{NL}\left( {\left| \psi \right|^2} \right)}}{{n_0}}\psi \left( {{\mathbf{x}},z} \right) = (K + V_L + V_{NL})\psi $$

which includes the kinetic term *K*, the linear index potential *V*_*L*_ from *δn*_*L*_(**x**), and the nonlinear index potential *V*_*NL*_ due to $\delta n_{NL}\left( {\left| \psi \right|^2} \right)$; *k*_0_ is the wavenumber of light in the medium. The above equation holds for both 1D and 2D photonic lattices. In 1D systems, the spatial coordinate is a scalar *x*, and in 2D systems, it is a vector ${\mathbf{x}} = x\hat x + y\hat y$. Here, we consider a 1D topological system; that is, we assume that the photonic lattice *V*_*L*_ can have nontrivial topological invariants. In our experiments and numerical simulations, we use the SSH lattice for *V*_*L*_(*x*). The photonic lattice and excitation scheme are illustrated in Fig. 1, where Fig. 1(a1) corresponds to a nontrivial lattice (Zak phase π) with two topological edge modes in the gap, and Fig. 1(c1) corresponds to a trivial lattice (Zak phase 0) without an edge state. In our theory, we use the above continuum model to describe the wave dynamics rather than its discrete version to obtain better correspondence with the experiments.

**Fig. 2:**
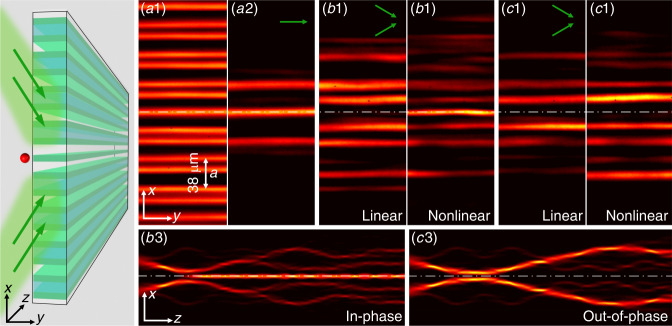
Nonlinearity-induced coupling into and “escaping” from the topological interface state. The illustration on the left shows two-beam tilted excitations (green arrows) of the topological defect from opposite directions. The top panels are from the experiment, where (a1) shows the cross-section of the lattice, (a2) shows the output of a topological interface state under single-beam (straight) excitation, and (b1) and (b2) show the outputs of two tilted in-phase beams ($k_{x} = \pm 1.4 \pi/a$) under linear and nonlinear excitation conditions. The bottom panel (b3) is from the simulation, showing a side view of the beam dynamics (up to a length of 40 mm) under nonlinear excitation. The right panels (c1–c3) have the same layout as (b1–b3) except that the defect is excited with two tilted out-of-phase beams. The white dashed-dotted line marks the position of the nontrivial interface defect channel in the SSH lattice in all figures

In our experiment, a 1D SSH photonic lattice, as illustrated in Fig. 1, is established by the *continuous-wave* (CW) laser-writing technique, which writes the waveguide lattice site-to-site in the bulk of a 20-mm-long nonlinear photorefractive crystal^60^. This technique allows a topological defect to be induced not only at the edge (Fig. 1(a1)) but also at the center, forming an interface (Fig. 2(a1)). Unlike femtosecond-laser writing in fused silica^61^, the lattice written in the nonlinear crystal is reconfigurable, so it can be readily changed from a trivial to a nontrivial structure in the same crystal. Once a chosen structure is written, it remains invariant during the period of experimental measurements (see “Methods”). In fact, since the SSH lattice is established here in a nonlinear crystal, it provides a convenient platform to investigate nonlinear wave dynamics in such a topological system, where the photorefractive nonlinear index potential *V*_*NL*_ is easily controlled by a bias field and the beam intensity^19,62^. Below, we demonstrate nonlinearity-induced coupling of light into topologically protected states in two different cases.

In the first case, the topological defect is located at the SSH lattice edge (Fig. 1, left panels). When a narrow stripe beam (FWHM 12 μm; input power 2.5 μW) is launched straight into the edge waveguide under linear conditions (the beam itself does not exhibit nonlinear self-action when the bias field is turned off), it evolves into a topological edge state (Fig. 1(a2)). Such an edge state, with a characteristic amplitude and phase populating only the odd-numbered waveguides counting from the edge, is topologically protected by the chiral symmetry of the SSH lattice^2^, as previously observed in the 1D photonic superlattice^43^. On the other hand, when the excitation is shifted away from the edge with a tilted broad beam to pump the defect ($k_x = 1.4\pi /a,$ where *a* = 38 μm is the lattice constant), we observe that the beam does not couple into the edge channel under linear conditions (Fig. 1b1). However, when the beam experiences a self-focusing nonlinearity (at a bias field of 160kV/m), a significant portion of the beam is coupled into the edge channel (Fig. 1b2), indicating that the nonlinearity somehow enables the energy to flow from the bulk modes into the topological edge mode of the SSH lattice. According to Eq. (1), we perform numerical simulation to examine the nonlinear beam dynamics using the parameters from the experiments, and the results are shown in Fig. 1(b3). We clearly see nonlinear coupling of the beam to the topological edge state of the SSH lattice, in agreement with the experiment.

For direct comparison, in the right panels of Fig. 1, we present the corresponding results obtained with the trivial SSH lattice. A dramatic difference is observed: (1) Under straight excitation, the input beam transports to quite a few waveguides close to the edge, but there is no dominant coupling to the first waveguide to form an edge state under linear conditions (Fig. 1(c2)). (2) For tilted excitation, however, the beam can easily enter the edge waveguide under linear conditions (Fig. 1(d1)), while it does not efficiently excite the edge waveguide within 20 mm of nonlinear propagation (Fig. 1(d2) and (d3)). Simulations for much longer distances beyond the crystal length indicate that the energy of the initial beam will eventually dissipate into the bulk under linear propagation. There is a key difference between trivial and nontrivial lattices under nonlinear propagation for tilted excitation: a distinct edge state persists in the nontrivial lattice, but no edge state exists in the trivial lattice. The underlying mechanism is analysed below in detail on the basis of nonlinear wave theory.

In the second case, the topological defect is located inside the SSH lattice (Fig. 2). To validate the nontrivial lattice established by laser writing, as shown in Fig. 2(a1), a single probe beam is launched straight into the defect channel, which leads to a topological interface state (Fig. 2(a2)). Then, two tilted beams are launched from opposite directions ($k_x = \pm 1.4\pi /a$) to pump the interface defect simultaneously, as illustrated in the left panel of Fig. 2. When the two beams are in-phase, light cannot couple into the defect channel in the linear condition (Fig. 2b1), but significantly enhanced coupling into the channel occurs in the nonlinear condition (Fig. 2(b2)). For comparison, similar experiments were performed on the same lattice under the same conditions except for two out-of-phase beams, which cannot couple into the defect channel under either linear or nonlinear excitation conditions (Fig. 2(c1) and (c2)). For linear excitation, topological protection prevents energy from flowing into the defect. For the nonlinear excitation, the nonlinear interaction of the two out-of-phase beams leads them to repel each other. This remarkable difference can be seen more clearly in the numerical simulation, where the nonlinearity-induced coupling (Fig. 2(b3)) and “repulsion” (Fig. 2(c3)) are evident. These results clearly show that optical beams from different directions can be pumped into a nontrivial defect channel due to optical nonlinearity under proper excitation conditions.

Now that we have presented our experiment and simulation results, which demonstrate nonlinear coupling into topologically protected states, we develop a general theoretical protocol for interpreting dynamics in nonlinear topological systems and employ it for our experiments. Let us assume that the linear component of the index of refraction *V*_*L*_(**x**) in Eq. (1) represents a topological photonic lattice, which is characterized by a topological invariant such as the Chern number for 2D lattices or the Zak phase for 1D lattices. The initial excitation is given by $\psi ({\mathbf{x}},z = 0)$. The subsequent propagation, governed by Eq. (1), gives us the complex amplitude of the electric field *ψ*(**x**, *z*) along the propagation direction, which in turn modulates the total index potential (linear and nonlinear) for any *z*: $V\left( {{\mathbf{x}},z} \right) = V_L\left( {\mathbf{x}} \right) + V_{NL}({\mathbf{x}},z)$. To determine and interpret the topological properties of the dynamically evolving nonlinear system, we use *the total index potential V*(**x**, *z*). The corresponding nonlinear eigenmodes $\varphi _{NL,n}\left( {{\mathbf{x}},z} \right)$ and nonlinear eigenvalues $\beta _{NL,n}(z)$ are defined by the equation:

2 $$(K + V_L + V_{NL})\varphi _{NL,n} = - \beta _{NL,n}\varphi _{NL,n}$$

We note that nonlinear eigenmodes and their eigenvalues are a function of the propagation distance *z* because nonlinear beam dynamics are generally not stationary. In contrast, the topological invariants of a linear system are drawn from the linear eigenmodes $\varphi _{L,n}({\mathbf{x}})$ with propagation constants *β*_*L*,*n*_, obtained from

3 $$(K + V_L)\varphi _{L,n} = - \beta _{L,n}\varphi _{L,n}$$

which are obviously not *z*-dependent. In both cases, *n* denotes the “quantum” numbers associated with the eigenmode, which can be associated with the Bloch wavevector and the band index for periodic photonic structures.

We emphasize several consequences of this approach: (i) The topological properties depend on the state of the system *ψ*(**x**, *z*) (this is natural because the system is nonlinear). These properties can be *inherited* from the underlying linear topological system, or they can *emerge* due to nonlinearity (see, e.g., Ref. ^24^). The *inherited* and *emergent* topological properties should be distinguished, as explained in the “Discussion” section below. (ii) The topological properties can change along the propagation direction. For example, we envision that for some initial conditions, the gap in the nonlinear spectrum $\beta _{NL,n}(z)$ could dynamically close and re-open, leading to a topological phase transition driven by nonlinearity. (iii) The evolution of the topological properties depends on the initial condition $\psi ({\mathbf{x}},z = 0)$. For a given initial condition, the subsequent dynamics yielding *V*(**x**, *z*) are unique.

**Fig. 3:**
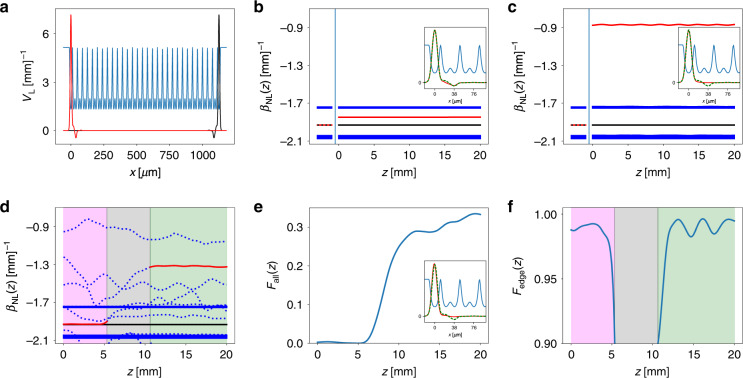
Nonlinear evolution of eigenvalues and coupling to the edge states in topologically nontrivial SSH lattices. **a** Two linear edge states (red and black) found in the SSH lattice (dark blue) used in our theoretical analysis. Band structure and nonlinearity-induced eigenvalue shifting under normal (straight) excitation conditions at low (**b**) and high (**c**) nonlinearity; the insets show the linear topological edge mode (green dashed line) and the nonlinear edge mode (red solid line). The evolving nonlinear eigenvalues $\beta _{NL,n}(z)$ are shown for *z* > 0. For comparison, the linear spectrum *β*_*L*,*n*_ is shown for *z* < 0. The red line is the eigenvalue of the (left) nonlinear edge mode, and the black line corresponds to the (right) linear edge mode, which is not excited. The thick blue lines are the bands. **d** Nonlinear eigenvalue evolution under tilted excitation conditions (corresponding to the left panels of Fig. 1). The red (black) line denotes the nonlinear (linear) edge eigenvalue as in **b**, **c**, while the individual blue dotted lines correspond to nonlinear localized states not inherited from the linear topological edge states. The three stages of the dynamics described in the text are denoted with the magenta, gray, and green shaded regions, respectively. **e** The overlap of the whole beam with the linear edge state *F*_all_(*z*). **f** The overlap of the linear and nonlinear edge modes *F*_edge_(*z*) at the three stages of evolution. See the text for details

Let us apply the protocol to interpret the dynamics observed in the experiment of Fig. 1 (left panel). The linear SSH lattice with *V*_*L*_(*x*) is in the topologically nontrivial regime, which has two degenerate edge states, as illustrated in Fig. 3a. The propagation constants of the linear eigenmodes *β*_*L*,*n*_ are illustrated in Fig. 3b, c (they are plotted in the region *z* < 0 for clarity, although they are *z*-independent); there are two bands corresponding to extended states, while the propagation constants of the localized edge states are in the middle of the gap as expected^41^. However, they are not at “zero energy” because we employ a continuous model with the experimental parameters. One can obtain the zero-energy states by adjusting the bottom of the linear potential through a transformation $V_L\left( x \right) \to V_L\left( x \right) + {\mathrm{constant}}$, but shifting the zero energy by a constant does not change the physics.

First, we analyse the initial excitation, which has the shape of the left edge state (colored red in Fig. 3a): $\psi \left( {{\mathbf{x}},z = 0} \right) = \sqrt {I_0} \varphi _{L,edge}$. This corresponds to the observation of Fig. 1a2. This linear edge state $\varphi _{L,edge}$ has a typical mode profile of topological characteristics: populating only odd-numbered waveguides with alternating opposite phases along the SSH lattice^44^. It is convenient to introduce the following quantities: (i) the edge state of the nonlinear system, $\varphi _{NL,edge}({\mathrm{x}},z)$, as the eigenmode of the potential *K* + *V*, which has the largest overlap with the linear edge state $\varphi _{L,edge}$, defined as $F_{edge}\left( z \right) = \left| {\left\langle {\varphi _{NL,edge}{\mathrm{|}}\varphi _{L,edge}} \right\rangle } \right|^2$; (ii) the overlap of the overall complex amplitude $\psi \left( {{\mathrm{x}},z} \right)$ with the linear edge state, defined as $F_{all}\left( z \right) = \left| {\left\langle {\psi \left( {{\mathrm{x}},z} \right){\mathrm{|}}\varphi _{L,edge}} \right\rangle } \right|^2/\left| {\left\langle {\psi {\mathrm{|}}\psi } \right\rangle } \right|^2$. The values of the overlaps *F*_edge_(*z*) and *F*_all_(*z*) are always between 0 and 1 by definition; the former tells us how similar the nonlinear and linear edge states are, and the latter tells us how much of the power of the beam populates the linear topological edge state.

In Fig. 3b, we show the eigenvalue evolution of the nonlinear system $\beta _{NL,n}(z)$ for a low nonlinearity (see the Supplementary Material for the calculation details and parameter values). The bands and the nonlinear eigenvalue of the right edge state (plotted for *z* > 0) are essentially identical to those of the linear spectrum $\beta _{L,n}$ (for comparison, $\beta _{L,n}$ is also plotted for *z* < 0, even though it is independent of *z*). However, the nonlinear eigenvalue $\beta _{NL,edge}$ of $\varphi _{NL,edge}$ (for the left edge state) is pushed towards the higher band, although it is still in the gap^34^. The nonlinear spectrum $\beta_{NL,n}(Z)$ is almost *z*-independent for this initial excitation. Our calculation shows that in this case, *F*_edge_(*z*) ≈ 0.99, while most of the power populates the left edge state, as *F*_all_(*z*) ≈ 0.99. The inset in Fig. 3b shows the profile of the topological linear edge state $\varphi_{L, edge},$, along with that of the nonlinear edge state $\varphi _{NL,edge}$ (at *z* = 15 mm). We see that the profile of the nonlinear edge state has the proper oscillations pertaining to the topological edge state, with the amplitude in odd waveguides (starting from the edge waveguide as the first one) and opposite phases in neighboring peaks. The edge state has an amplitude mainly in the first (edge) waveguide and then the third waveguide. If the nonlinearity is increased above some threshold value, the nonlinear eigenvalue $\beta _{NL,edge}$ moves across the band to appear above the first band, as illustrated in Fig. 3c. From the mode profiles shown in the inset of Fig. 3c, we find that the nonlinear edge state is essentially identical to the linear one in the edge waveguide, but it lacks an amplitude in the third waveguide. This difference is more easily seen when we use a larger lattice coupling than the one obtained from the experimental parameters.

We conclude that for the initial excitation $\psi \left( {{\mathbf{x}},z = 0} \right) = \sqrt {I_0} \varphi _{L,edge}$, when $\beta _{NL,edge}$ is in the gap, the localization is induced by the topology, and the nonlinear edge state can be regarded as a topological edge state. When $\beta _{NL,edge}$ is above the upper band (in the semi-infinite gap), the localization is induced by nonlinearity. Even though the mode profile in the edge channel is *inherited* from the linear topological system (see the inset in Fig. 3c), due to the lack of mode features in the third waveguide, the nonlinear edge mode should not be characterized as topological when $\beta_{NL,edge}$ is in the semi-infinite gap. A related analysis of similar scenarios can be found in Refs. ^30,59^.

A theoretical analysis of the experiments corresponding to tilted excitation in Fig. 1(b2) is more involved because in this case, the dynamics are far from stationary, yet this case captures the essence of the theoretical protocol. The beam is launched at *x* = 1.2*a* at an angle $k_{x}=-1.4 \pi/a$ towards the edge located at *x* = 0 (see Fig. 3a). For this initial excitation, *F*_all_(*z* = 0) ≈ 0; i.e., at the input of the medium, the beam does not excite the linear edge state. Figure 1(b1) is easily understood, as *F*_all_(*z*) is *z*-independent in the linear dynamics. The evolution of the nonlinear spectrum $\beta _{NL,n}(z)$ is depicted in Fig. 3d. First, we note that the band structure (thick blue lines) corresponding to the bulk states is essentially *z*-invariant and is equivalent to that of the linear system. Due to the self-focusing nonlinearity, the dynamics are manifested in the localized modes of $V\left( {x,z} \right) = V_L\left( x \right) + V_{NL}(x,z)$; there are quite a few evolving localized modes of *V*(*x*,*z*), with eigenvalues $\beta _{NL,n}(z)$ indicated by the dotted blue lines in Fig. 3d. We focus only on the nonlinear edge state $\varphi _{NL,edge}$ and its eigenvalue $\beta _{NL,edge}$, plotted in Fig. 3d with a solid red line. From Fig. 3e, f, which illustrate *F*_all_(*z*) and *F*_edge_(*z*), respectively, we see that the dynamics can be divided into three stages. More specifically, the sudden drop of *F*_edge_(*z*) at *z* = 5 mm indicates the end of the first stage, while the sudden increase at *z* = 11 mm indicates the end of the second stage of the dynamics (see Fig. 3f). In the first stage (shaded magenta in Fig. 3d, f), the launched beam travels towards the edge, and the edge state is not populated, as $F_{all}(z) \approx 0$; consequently, $\beta _{NL,edge}$ is in the gap (see the left red line in Fig. 3d), and *F*_edge_(*z*) is close to unity. In the second stage (shaded gray), when the beam is at the edge, the linear edge state becomes populated, and *F*_all_(*z*) increases. In this stage, the beam strongly perturbs the local structure of the lattice at the edge, as seen from the drop in *F*_edge_(*z*) in Fig. 3f, which means that none of the nonlinear localized states are similar to $\varphi _{L,edge}$ (thus, none of the nonlinear eigenvalues is colored red in Fig. 3d in the second stage). In the third stage (shaded green), a large portion of the beam is reflected, but ~30% of the beam becomes trapped in a localized edge state: *F*_all_(*z*) ≈ 0.3, as shown in Fig. 3e. There is a well-defined nonlinear edge state with eigenvalue $\beta _{NL,edge}$ above the first band, not in the gap, as indicated by the right red line in Fig. 3d. The profile of this nonlinear edge state is mostly inherited from the topology of the linear structure, as seen in the inset of Fig. 3e and the overlapping *F*_edge_(*z*) ≈ 0.98 shown in Fig. 3f; however, it lacks the topological mode feature in the third waveguide. We conclude that the localization is dominantly induced by nonlinearity. We should emphasize that the linear edge state does not continuously transform into the nonlinear edge state during propagation, because of the strong deformation of the lattice in the second stage of the dynamics. After this distortion, one of the localized states from stage two re-emerges as the new nonlinear localized edge state in stage three, as can be traced by following the nonlinear eigenmodes alongside the *F*-functions plotted in Fig. 3d, e.

The details of the theoretical analysis corresponding to the right panel of Fig. 1 (for the SSH lattice in the topologically trivial regime) and Fig. 2 (for excitation of the topological defect with two beams) are shown in the Supplementary Material and summarized here. The results in the right panels of Fig. 1 can be interpreted as follows: all the linear modes are extended, as the SSH lattice is in the topologically trivial regime. The beam initially excites many of these states. In the linear regime illustrated in Fig. 1(d1), the beam approaches the waveguide at the edge and then travels along the edge for the length of the crystal. In other words, for short propagation distances (smaller than the length of the crystal), the phases of all linearly excited (extended) modes add together such that the intensity of the beam populates the waveguides close to the edge in Fig. 1(d1). However, for a very long propagation distance, due to the de-phasing of the excited bulk modes, the beam will spread into the lattice. In the nonlinear case corresponding to Fig. 1(d2), (d3), the nonlinearity creates evolving localized states, which are not related to the topological origin, as none of the nonlinear modes resemble the linear topological edge state. In fact, in this trivial lattice structure, the localized modes arise purely due to the nonlinear index change, as is typically the case with optical solitons. A light beam forms a few self-trapped filaments around these states and evolves in this fashion for the propagation distance of the crystal length in the experiment. As the initial excitation is not at the edge, the location of the self-trapped filaments is also not at the edge, as illustrated in Fig. 1(d2), (d3).

Regarding the excitation of the defect mode with two beams at opposite angles, when the beams are in phase, there are again three stages of the dynamics, which are equivalent to those shown in Fig. 3d–f. In the first stage, the beams travel towards the linear defect channel in the center of the lattice; the defect state is not yet populated, and its eigenvalue is in the gap. Many evolving nonlinear localized states arise due to nonlinearity but not to topology. In the second stage, the linear defect state starts to become populated, but the lattice is distorted locally due to nonlinear action, so none of the nonlinear states are similar to the linear defect state. In the third stage, some of the incident light (~20–30% for the parameters used here) is trapped in the defect state, while the rest is repelled. There is a well-defined nonlinear defect state with a profile in the defect channel inherited from the linear defect state and a nonlinear eigenvalue emerging above the first band. Thus, conceptually, an identical scenario to that shown in Fig. 3d–f occurs. The difference is that the defect state can now be coupled from both sides, and this could be extended to coupling light from all directions in a 2D SSH-type system, leading to a nonlinear “tapered” topological waveguide. Such potential applications certainly merit further research.

When the two incident input beams at opposite angles are out of phase, again there are three stages of the dynamics analogous to those presented above (see the Supplementary Material). However, the linear defect state is not populated by any of them. The eigenvalue of the nonlinear defect state is within the gap in the first and third stages of the dynamics. In the second stage, when the light is close to the defect state, the lattice structure is distorted, and none of the nonlinear localized states are very similar to the linear defect state. In fact, the two beams stay away from each other, and the defect in this case is related to the nonlinear interaction of out-of-phase soliton-like beams rather than to topology.

## Discussion

The interplay of nonlinearity and topology is somewhat analogous to the interplay of locality and globality, as most of the studied optical nonlinearities are local, and the topology describes the global properties of a system. To analyse nonlinear topological systems, one must find an appropriate way to connect the local and global properties of the underlying systems. The proposed theoretical protocol does just that: it takes the total change in the index potential $V\left( {{\mathbf{x}},z} \right) = V_L\left( {\mathbf{x}} \right) + V_{NL}({\mathbf{x}},z)$ (which includes the nonlinear term), and analyses the topological properties of the nonlinear system.

Our theory is designed to unravel non-stationary dynamics, which are at the heart of the nonlinear coupling presented here. In this case, the potential $V\left( {x,z} \right) = V_L\left( x \right) + V_{NL}(x,z)$ evolves along *z* (*z* is the “time” in our system), and the topological quantities can in principle change during the evolution. In the specific lattice system studied above, the gap in the nonlinear spectrum $\beta _{NL,n}(z)$ does not close at any *z*, and the bands remain fairly intact in the presence of nonlinearity. However, we observe that the interplay of nonlinearity and topology can couple light into the topological edge state of the linear system, which is inadmissible for entirely linear dynamics (e.g., see Fig. 1(b2) and Fig. 3d–f). When this happens, we can identify the nonlinear edge mode $\varphi _{NL,edge}$, which inherits the profile of the linear edge mode $\varphi _{L,edge}$ in the edge channel and is quantified by *F*_edge_(*z*) ≈ 0.98 after the nonlinear coupling has occurred, although it lacks the amplitude in the third waveguide. Thus, for a high nonlinearity, the eigenvalue of $\varphi _{NL,edge}$ moves outside the gap (see Fig. 3c), and the edge mode is dominated by nonlinearity but has some features inherited from the linear topological edge mode; for a low nonlinearity, its eigenvalue stays inside the gap, so it is dominated mainly by the topology. For the other initial conditions studied in the experiment, presented in Fig. 2, the interplay of topology and nonlinearity is conceptually the same.

Let us comment on the calculation of topological invariants in finite nonlinear lattices. Topological invariants for periodic lattices, the Chern number for 2D lattices and the Zak phase for 1D lattices, are calculated for an infinite periodic system by integrating over the Brillouin zone^2^. These invariants, in a finite lattice, are manifested by edge states (edge modes), such as those in the SSH model. When we deal with a finite nonlinear system, we cannot straightforwardly use the formulae for calculating the Chern number and the Zak phase for infinite periodic systems. This problem has already been addressed in the literature; see, e.g., Refs. ^63,64^, where the so-called Bott index was calculated. Here, we develop an approach that explores nonlinear eigenmodes and compares them with the relevant linear eigenmodes. This approach relies on the following facts: (i) the Zak phase (as well as the Chern number) is calculated from the eigenmodes, and (ii) for a linear system, the Zak phase is well known in the topologically trivial or nontrivial regime. Thus, we focus on how the eigenmodes change when we introduce nonlinearity. This is quantified by the overlaps of the linear and nonlinear modes and shown in the mode profiles and the positions of their eigenvalues in the spectrum.

Before closing, let us discuss the distinction between *inherited* and *emergent* nonlinear phenomena. If the underlying nonlinear system is topological, this distinction is manifested in the topological invariants pertinent to the edge modes of the system and perhaps in other quantities. During nonlinear evolution, some of the local quantities, such as the edge modes, can be modified by nonlinearity without closing the gap or changing the topological invariants. If these modified nonlinear modes are similar according to some measure (such as the quantity *F*_edge_ used here) to the modes of the underlying linear system, we say that their properties are *inherited*. However, if the underlying linear system is initially topologically trivial, under some conditions, it may happen that the nonlinear dynamics change the topological invariants and turn the system into a topologically nontrivial regime. Because the action of nonlinearity is normally local, for such a scenario to occur, it appears that the excitation must be extended. For this type of scenario, which is in principle possible, we say that the topological properties of the nonlinear system are *emergent* because they are not present in the corresponding linear system. Although emergent nonlinear phenomena such as band inversion and topological phase transitions have not been observed in the particular setting employed in this study, we believe that they should exist in some nonlinear topological systems.

In conclusion, we have established trivial and nontrivial photonic SSH lattices by direct cw-laser writing in a bulk nonlinear crystal and thereby experimentally demonstrated the nonlinearity-induced coupling of light into a topological edge state. In particular, we have shown that two optical beams from different directions can couple into (stay away from) a nontrivial defect channel under nonlinear (linear) excitation upon collision. We have developed a theoretical protocol to explain the dynamics observed in this lattice system. Our theory shows that, by nonlinear excitation of bulk modes, depending on the input power (i.e., the strength of the nonlinearity), the trapped light beam can evolve into a nonlinear edge mode with a profile featuring a topological edge state fully inherited from the underlying linear system. These features exemplify the interplay of topology and nonlinearity in topologically nontrivial systems. The protocol presented in this work is general, applicable not only to non-stationary and dynamically evolving systems such as the one studied here but also to systems other than SSH lattices and even to systems beyond the photonic platform.

For future research, we envision that many fundamental issues could arise from systems with *emergent*, rather than solely *inherited*, nonlinear topological phenomena, where nonlinear dynamics can close and reopen the gap and induce topological phase transitions. A toolkit for such studies is presented here. Our results may bring about insights and advances in the nonlinear control of topological quantum states in similar systems^28,36,54,55^ as well as in photonic parity-time-symmetric and anomalous Floquet topological systems where the excitation can be tuned by nonlinearity^48,65^.

## Materials and methods

**Fig. 4:**
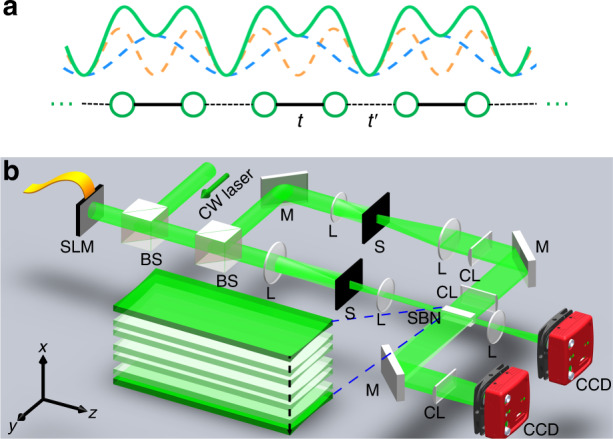
Experimental scheme for laser-writing photonic SSH lattices in a nonlinear crystal. **a** Illustration of the SSH model, where *t* and *t*′ represent strong and weak coupling, respectively. The green solid curve shows the SSH lattice formed by superimposing the two periodic lattices depicted by the dashed curves. **b** Experimental setup for writing and probing the SSH lattice. SLM spatial light modulator, BS beam splitter, M mirror, L circular lens, S single slit, CL cylindrical lens, SBN strontium barium niobite crystal, CCD camera. The upper path is for the lattice-writing beam (ordinarily polarized), and the lower path is for the probe beam (extraordinarily polarized). The lattice structure in the crystal is magnified and shown in the lower inset

Our experimental method for laser-writing the 1D SSH photonic lattice is shown in Fig. 4, where Fig. 4a illustrates the idea of establishing the superlattice by overlapping two periodic index potentials of different periods^43^ and Fig. 4b shows the experimental setup. The two periodic potentials are indicated by the dashed curves in Fig. 4a, which are written into the nonlinear SBN crystal one after another due to the optically induced local index change. Superposing these two potentials produces the SSH lattice (solid curve), where the coupling of neighboring sites can be fine-tuned by shifting their relative position. When the lattice is terminated at the strong-coupling “bond” denoted by *t*, this corresponds to the nontrivial case shown in the left panels of Fig. 1, since in this case the intra-cell coupling is weaker than the inter-cell coupling. The opposite case occurs when the lattice is terminated at the weak-coupling “bond” denoted by *t*′, which represents the trivial lattice shown in the right panels of Fig. 1. Since the cw-laser writing technique^60^ is used to induce the potential one by one, the lattice edge (and interface) can be readily reconfigured by this method.

In the setup of Fig. 4b, the upper and lower paths correspond to the lattice-writing and probing beams, respectively. A collimated laser beam (with wavelength 532 nm and power 100 mW) illuminates a programmable spatial light modulator (SLM), which alternatively generates the writing and probing beams. In the writing path, the beam exiting the SLM is collimated and spatially filtered with a narrow single slit and then further compressed into a narrow stripe beam with an FWHM of ~10 μm by a pair of cylindrical lenses, so it is long enough to cover the entire 20-mm-long SBN:61 crystal. Its input position to the crystal is precisely controlled by the SLM. Through a multi-step writing process in the biased crystal (with an applied field 240 kV/m), the desired SSH lattice is established with a lattice constant of 38μm. Because of the “memory” effect of the photorefractive crystal, this index lattice remains intact for more than one hour, enough time to measure the beam dynamics. In the lower path, the probe (stripe) beam is launched into the lattice, and its input size, position, and direction can all be adjusted by the SLM. In addition, the probe beam can undergo linear or nonlinear propagation through the lattice, depending on whether or not a proper bias field is applied^62^. The CCD camera in the writing beam path is used to examine the position of the stripe writing beam, and the other CCD is used to monitor the input and output of the probe beam propagating through the lattice. To image a particular SSH photonic lattice after it is written, a single stripe beam is launched into the crystal to probe the waveguides one by one, and then all guided outputs of the probe beam are superimposed to obtain the lattice structure of Fig. 1(a1), (c1).

## Supplementary information

Supplementary Material

## References

[1] Lu L, Joannopoulos JD, Soljačić M (2014). Topological photonics. Nat. Photonics.

[2] Ozawa T (2019). Topological photonics. Rev. Mod. Phys..

[3] Klitzing KV, Dorda G, Pepper M (1980). New method for high-accuracy determination of the fine-structure constant based on quantized Hall resistance. Phys. Rev. Lett..

[4] Thouless DJ (1982). Quantized Hall conductance in a two-dimensional periodic potential. Phys. Rev. Lett..

[5] Kane CL, Mele EJ (2005). Z_2_ Topological order and the quantum spin Hall effect. Phys. Rev. Lett..

[6] Kane CL, Mele EJ (2005). Quantum spin Hall effect in graphene. Phys. Rev. Lett..

[7] Qi XL, Zhang SC (2011). Topological insulators and superconductors. Rev. Mod. Phys..

[8] Haldane FDM, Raghu S (2008). Possible realization of directional optical waveguides in photonic crystals with broken time-reversal symmetry. Phys. Rev. Lett..

[9] Raghu S, Haldane FDM (2008). Analogs of quantum-Hall-effect edge states in photonic crystals. Phys. Rev. A.

[10] Hatsugai Y (1993). Chern number and edge states in the integer quantum Hall effect. Phys. Rev. Lett..

[11] Jackiw R, Rebbi C (1976). Solitons with fermion number 1/2. Phys. Rev. D..

[12] Wang Z (2009). Observation of unidirectional backscattering-immune topological electromagnetic states. Nature.

[13] Rechtsman MC (2013). Photonic Floquet topological insulators. Nature.

[14] Hafezi M (2013). Imaging topological edge states in silicon photonics. Nat. Photonics.

[15] Khanikaev AB (2013). Photonic topological insulators. Nat. Mater..

[16] Tsui DC, Stormer HL, Gossard AC (1982). Two-dimensional magnetotransport in the extreme quantum limit. Phys. Rev. Lett..

[17] Fermi, E. et al. *Studies of the Nonlinear Problems*. (Los Alamos Scientific Lab, 1955).

[18] Pierangeli D (2018). Observation of Fermi-Pasta-Ulam-Tsingou recurrence and its exact dynamics. Phys. Rev. X.

[19] Malkova N (2009). Transition between Tamm-like and Shockley-like surface states in optically induced photonic superlattices. Phys. Rev. A.

[20] Manela O (2010). Hofstadter butterflies in nonlinear Harper lattices, and their optical realizations. N. J. Phys..

[21] Lumer Y (2013). Self-localized states in photonic topological insulators. Phys. Rev. Lett..

[22] Chen SQ (2014). Broadband optical and microwave nonlinear response in topological insulator. Optical Mater. Express.

[23] Leykam D, Chong YD (2016). Edge solitons in nonlinear-photonic topological insulators. Phys. Rev. Lett..

[24] Hadad Y, Khanikaev AB, Alù A (2016). Self-induced topological transitions and edge states supported by nonlinear staggered potentials. Phys. Rev. B.

[25] Bleu O, Solnyshkov DD, Malpuech G (2016). Interacting quantum fluid in a polariton Chern insulator. Phys. Rev. B.

[26] Zhou X (2017). Optical isolation with nonlinear topological photonics. N. J. Phys..

[27] Kartashov YV, Skryabin DV (2017). Bistable topological insulator with exciton-polaritons. Phys. Rev. Lett..

[28] Mittal S, Goldschmidt EA, Hafezi M (2018). A topological source of quantum light. Nature.

[29] Shalaev MI (2019). Robust topologically protected transport in photonic crystals at telecommunication wavelengths. Nat. Nanotechnol..

[30] Dobrykh DA (2018). Nonlinear control of electromagnetic topological edge states. Phys. Rev. Lett..

[31] Smirnova D (2020). Nonlinear topological photonics. Appl. Phys. Rev..

[32] Solnyshkov DD (2017). Chirality of topological gap Solitons in Bosonic dimer Chains. Phys. Rev. Lett..

[33] Marzuola, J. L. et al. Bulk Soliton dynamics in Bosonic topological insulators. https://arxiv.org/abs/1904.10312 (2019).

[34] Smirnova DA (2019). Topological edge states and gap solitons in the nonlinear Dirac model. Laser Photonics Rev..

[35] Kruk S (2019). Nonlinear light generation in topological nanostructures. Nat. Nanotechnol..

[36] Wang Y (2019). Topologically enhanced harmonic generation in a nonlinear transmission line metamaterial. Nat. Commun..

[37] Smirnova D (2019). Third-harmonic generation in photonic topological Metasurfaces. Phys. Rev. Lett..

[38] St-Jean P (2017). Lasing in topological edge states of a one-dimensional lattice. Nat. Photonics.

[39] Bahari B (2017). Nonreciprocal lasing in topological cavities of arbitrary geometries. Science.

[40] Bandres MA (2018). Topological insulator laser: experiments. Science.

[41] Su WP, Schrieffer JR, Heeger AJ (1979). Solitons in Polyacetylene. Phys. Rev. Lett..

[42] Zak J (1989). Berry’s phase for energy bands in solids. Phys. Rev. Lett..

[43] Malkova N (2009). Observation of optical Shockley-like surface states in photonic superlattices. Opt. Lett..

[44] Keil R (2013). The random mass Dirac model and long-range correlations on an integrated optical platform. Nat. Commun..

[45] Xiao M, Zhang ZQ, Chan CT (2014). Surface impedance and bulk band geometric phases in one-dimensional systems. Phys. Rev. X.

[46] Blanco-Redondo A (2016). Topological optical waveguiding in silicon and the transition between topological and trivial defect states. Phys. Rev. Lett..

[47] Kruk S (2017). Edge states and topological phase transitions in Chains of dielectric nanoparticles. Small.

[48] Weimann S (2017). Topologically protected bound states in photonic parity–time-symmetric crystals. Nat. Mater..

[49] Saei Ghareh Naz E (2018). Topological phase transition in a stretchable photonic crystal. Phys. Rev. A.

[50] Poddubny A (2014). Topological Majorana states in zigzag chains of plasmonic nanoparticles. ACS Photonics.

[51] Bleckmann F (2017). Spectral imaging of topological edge states in plasmonic waveguide arrays. Phys. Rev. B.

[52] Kitagawa T (2012). Observation of topologically protected bound states in photonic quantum walks. Nat. Commun..

[53] Cardano F (2017). Detection of Zak phases and topological invariants in a chiral quantum walk of twisted photons. Nat. Commun..

[54] Blanco-Redondo A (2018). Topological protection of biphoton states. Science.

[55] Bello M (2019). Unconventional quantum optics in topological waveguide QED. Sci. Adv..

[56] Zhao H (2018). Topological hybrid silicon microlasers. Nat. Commun..

[57] Parto M (2018). Edge-mode lasing in 1D topological active arrays. Phys. Rev. Lett..

[58] Han C (2019). Lasing at topological edge states in a photonic crystal L3 nanocavity dimer array. Light. Sci. Appl..

[59] Bisianov A (2019). Stability of topologically protected edge states in nonlinear fiber loops. Phys. Rev. A.

[60] Xia SQ (2018). Unconventional flatband line states in photonic Lieb lattices. Phys. Rev. Lett..

[61] Szameit A (2005). Discrete nonlinear localization in femtosecond laser written waveguides in fused silica. Opt. Express.

[62] Fleischer JW (2003). Observation of two-dimensional discrete solitons in optically induced nonlinear photonic lattices. Nature.

[63] Bandres MA, Rechtsman MC, Segev M (2016). Topological photonic quasicrystals: fractal topological spectrum and protected transport. Phys. Rev. X.

[64] Loring TA, Hastings MB (2010). Disordered topological insulators via C^*^-algebras. EPL (Europhys. Lett.).

[65] Mukherjee S, Rechtsman MC (2020). Observation of Floquet solitons in a topological bandgap. Science.

