# Supplementary material for: Nontrivial coupling of light into a defect: the interplay of nonlinearity and topology
Source: Light Sci Appl. 2020 Aug 19;9:147. doi: 10.1038/s41377-020-00371-y (PMC7438503; doi:10.1038/s41377-020-00371-y)
Supplement: Supplementary file 1 — Supplementary Material [file 41377_2020_371_MOESM1_ESM.pdf]

Supplementary Material:

Nontrivial coupling of light into a defect: the interplay of nonlinearity and topology

Shiqi Xia[1+], Dario Jukić[2+], Nan Wang[1+], Daria Smirnova[3], Lev Smirnov[4], Liqin Tang[1,5], Daohong Song[1,5], Alexander Szameit[6], Daniel Leykam[7,8], Jingjun Xu[1,5], Zhigang Chen[1,5,9], and Hrvoje Buljan[1,10]

[1] The MOE Key Laboratory of Weak-Light Nonlinear Photonics, TEDA Applied Physics Institute and School of Physics, Nankai University, Tianjin 300457, China

[2] Faculty of Civil Engineering, University of Zagreb, A. Kačića Miošića 26, 10000 Zagreb, Croatia

[3] Nonlinear Physics Centre, Research School of Physics, Australian National University, Canberra ACT 2601, Australia

[4] Institute of Applied Physics, Russian Academy of Science, Nizhny Novgorod 603950, Russia

[5] Collaborative Innovation Center of Extreme Optics, Shanxi University, Taiyuan, Shanxi 030006, People's Republic of China

[6] Institut für Physik, Universität Rostock, Albert-Einstein-Strasse 23, 18059 Rostock, Germany

[7] Center for Theoretical Physics of Complex Systems, Institute for Basic Science (IBS), Daejeon 34126, Korea

[8] Basic Science Program, Korea University of Science and Technology, Daejeon 34113, Korea

[9] Department of Physics and Astronomy, San Francisco State University, San Francisco, California 94132, USA

[10] Department of Physics, Faculty of Science, University of Zagreb, Bijenička c. 32, 10000 Zagreb, Croatia

[+]These authors made equal contribution.

songdaohong@nankai.edu.cn, hbuljan@phy.hr, zgchen@nankai.edu.cn

Details of theoretical analysis

1. Edge excitation with a single beam in nontrivial SSH lattices

Details of the theoretical analysis for Fig. 3 in the main text are presented here, corresponding to edge excitation with a single input beam. The total potential (linear and the nonlinear terms), corresponding to the photorefractive medium used in experiments is

$$V(x,z) = \frac{k_0}{n_0}\frac{\Delta n}{1+i_L(x)+i_{NL}(x,z)}, \tag{1}$$

where n_0=2.35, $k_0 = \frac{2\pi n_0}{\lambda}$, $\lambda = 532$ nm, $\Delta n = 4.36\times 10^{-4}$;

$$i_L(x) = N_L\left[0.8\left(\cos(\frac{\pi(x-c)}{a})\right)^2 + \left(\cos(\frac{\pi(x-c-a/4)}{a/2})\right)^2\right], \tag{2}$$

where a =38 μm is the lattice constant, c is offset constant so that the left edge is centered at $x=0$, and the normalization N_L is such that the lattice amplitude (the maximum value of $i_L(x)$) is 2.88. The nonlinear contribution is

$$i_{NL}(x) = \gamma|\psi(x,z)|^2, \tag{3}$$

where γ tunes the intensity of the beam without changing its shape or the bias field (and therefore the strength of the nonlinear index change). The quantities $i_L(x)$ and $i_{NL}(x)$ are dimensionless. For the SBN crystal used in the experiments, the nonlinear index change parameter is $\Delta n = -n_0^3 r_{33} E_0/2$, where r_{33} is the effective electro-optic coefficient of the SBN crystal, and E_0 is the bias field. The strength and the sign of the bias field determines the strength and the sign of the nonlinearity. When the bias field is zero, i.e., $E_0 = 0$, the system is linear.

The initial states for Figs. 3(b, c) are identical in shape to the linear edge state, $\psi(\mathbf{x}, z = 0) = \sqrt{I_0}\varphi_{L,edge}$. For a low nonlinearity [Fig. 3(b)], we choose $\gamma \max|\psi(\mathbf{x}, 0)|^2$ =0.30, and for a high nonlinearity [Figs. 3(c-f)], we set $\gamma \max|\psi(\mathbf{x}, 0)|^2 = 12.5$. At the critical value of $\gamma \max|\psi(\mathbf{x}, 0)|^2$ =0.72, the nonlinear eigenvalue $\beta_{NL,edge}$ moves from inside the gap [Fig. 3(b)] to above the first band [Fig. 3(c)].

The shape of the initial state for simulations (mimicking the input broad beam for lattice excitation used in experiment) in Figs. 3(d, e) is given by

$$\psi(x, z = 0) = \sqrt{I_0} \exp(\frac{-i1.4\pi x}{a})\exp(-\frac{(x-45.6\ \mu\text{m})^2}{(22.8\ \mu\text{m})^2}). \tag{4}$$

The strength of the nonlinearity is set to γI_0 =4.10.

2. **Interface excitation with two beams in nontrivial SSH lattices**

Here we provide theoretical analysis of experiments and numerical simulations presented in Fig. 2 of the main text. The theoretical protocol has already been applied in the main text to obtain Fig. 3 and to explain the experimental results of Fig. 1.

The outline of Figs. S1(a-c) is identical to that of Figs. 3(a-c) in the main text, except now it is for the SSH lattice with an interface defect located in the center of the lattice. In Fig. S1(a) we show the linear SSH lattice which has three localized states in the band gap: the left (red) and the right (black) edge states, and the interface (magenta) defect state. When the input excitation is chosen to match the shape of the (linear) defect state, $\psi(\mathbf{x}, z = 0) = \sqrt{I_0}\varphi_{L,defect}$, for a low nonlinearity $\gamma \max|\psi(\mathbf{x}, 0)|^2 = 0.312$, the eigenvalue of the defect state $\beta_{NL,defect}(z)$ is shifted towards the first band due to nonlinearity but remains inside the gap, as shown in Fig. S1(b). We see that

$\beta_{NL,defect}(z)$ essentially stays constant during the propagation. When the nonlinearity is increased to a threshold value, $\beta_{NL,defect}(z)$ is found to be above the first band, as illustrated in Fig. S1(c) for $\gamma \max|\psi(\mathbf{x},0)|^2 = 13.0$.

Now we discuss the scenario in which two tilted beams are sent from opposite angles towards the interface defect site, as described in Fig. 2 of the main text. In this example we use the same parameters as that for Figs. 3(d-f) of the main text, that is, the same structure of the input beams described by Eq. (4) above (one from left, and the other from right of the defect). In Figs. S1(d, f) we show results for the case when two beams are in phase, and corresponding results for the out-of-phase beams are shown in Figs. S1(g-i). The layout of Figs. S1(d-f) [and S1(g-i)] is identical to that of Fig. 3(d-f) in the main text, with three shaded regions (magenta, gray, and green) corresponding to three different stages of nonlinear beam dynamics.

When two beams are in phase, we again have three stages of the dynamics analogous to those from Fig. 3 in the main text. The evolution of the nonlinear eigenvalue spectrum $\beta_{NL,n}(z)$ is presented in Fig. S1(d). In the first stage (shaded in magenta), the wavepacket has not yet arrived at the defect site, and the eigenvalue $\beta_{NL,defect}(z)$ of the nonlinear defect state $\varphi_{NL,defect}$ is the same as in the linear case, as denoted by the solid magenta line. In addition to two edge states and the defect state elaborated so far, we observe additional localized states which appear due to nonlinearity (and not topology of the linear lattice); their nonlinear eigenvalues are indicated with blue dotted lines. In the first stage, the beam does not excite the defect state, i.e., the overlap $F_{all}(z) = \left|\langle\psi(\mathrm{x},z)|\varphi_{L,defect}\rangle\right|^2/|\langle\psi|\psi\rangle|^2$ is close to zero, as illustrated in Fig. S1(e). Consequently, in this first stage $F_{defect}(z) = \left|\langle\varphi_{NL,defect}|\varphi_{L,defect}\rangle\right|^2$ is approximately one, as shown in Fig. S1(f). In the second stage (shaded grey), the two beams have arrived at the defect site, which changes significantly the local structure of the lattice. None of the nonlinear eigenmodes is similar to the linear defect state $\varphi_{L,defect}$, as can be seen from the drop of $F_{defect}(z)$ illustrated in Fig. S1(f). In this stage, the linear defect state becomes populated, see Fig. S1(e). In the third stage (shaded green), the wavepacket is partially reflected, but a part of it stays at the defect site (about 20-30% as can be seen from Fig. S1(e)). The nonlinear defect state $\varphi_{NL,defect}$ has again a great overlap with the linear defect state $\varphi_{L,defect}$ [Fig. S1(f)], but its propagation constant $\beta_{NL,defect}(z)$ is now above the first band [Fig. S1(d)].

Figures S1(g-i) illustrate results obtained for the two beams initially out of phase. Evolution of the nonlinear spectrum is shown in Fig. S1(g). In sharp contrast to the in-phase case, the defect eigenvalue $\beta_{NL,defect}(z)$ remains inside the gap at all stages, as seen from Fig. S1(g). The overlap of the whole beam with the linear defect state $F_{all}(z) \approx 0$ at all times, as seen from Fig. S1(h), indicating that coupling to the defect state does not occur. From Fig. S1(i), we see that as the beams approach the defect, $F_{defect}(z)$ decreases. This occurs due to the change of the local index of refraction, i.e., the local distortion of the lattice. In this case the coupling does not occur, and $F_{defect}(z)$ revives after the beams are repelled from each other at the defect.

3. Edge excitation with a single beam in trivial SSH lattices

For completeness and direct comparison, in Fig. S2 we present a detailed theoretical analysis corresponding to the excitation of the trivial SSH lattice; pertinent experiments are presented in the right panel of Fig. 1 in the main text. The layout of Fig. S2, and the parameters used such as the nonlinearity strength, are identical to those for Fig. 3 in the main text.

In Fig. S2(a), we show the trivial SSH lattice used in the simulations; there are no topological edge or defect states. In Fig. S2(b, c), we show dynamics of the nonlinear eigenvalues for the initial excitation which has the shape of the topological edge state of the nontrivial SSH lattice: $\psi(\mathbf{x}, z = 0) = \sqrt{I_0}\varphi_{L,edge}$ (identical initial condition as for Fig. 3(b, c)). We observe that dynamics of the bands is practically z-independent (thick blue lines); the bands are the same as for the underlying linear system. In Fig. S2(b) we see that there are two nonlinear localized states, one with the eigenvalue in the semi-infinite gap above the first band (solid red line), and the other with the eigenvalue in the gap (dotted red line). In Fig. S2(c) we again see two localized states, with eigenvalues above the first band. For this initial excitation, most of the beam power is present in the two eigenmodes induced by the nonlinearity (no feature of topology is present), which evolves along the propagation axis.

In Fig. S2(d, f), we show results for the excitation at an angle towards the edge of the trivial lattice. Figure S2(d) illustrates dynamics of nonlinear eigenvalue spectrum. We see the bands which are z-independent (thick blue lines). Dynamics is again manifested in the evolution of nonlinearly excited localized eigenmodes, whose propagation constants are illustrated with dotted blue lines. During the evolution, none of them resembles the topological linear defect state of the nontrivial SSH lattice, which follows from Fig. S2(f) displaying $F_{edge}(z)$; we only see an occasional rise of the overlap above 70%; this occurs due to the nonlinear mode beating, but it does not persist and cannot reach high values to be related to the topological edge state in any way (as compared to 98-99% overlap observed in Fig. 3 of the topologically nontrivial lattice reported in the main text); it does not make sense to display smaller overlaps as they carry no meaning subject to interpretation. From Fig. S2(e), we see that the overlap of the beam with the topological edge state of the linear system is always small, indicating that this is not a topological phenomenon as those occurring in the nontrivial lattice, but rather it is due to the nonlinearity.

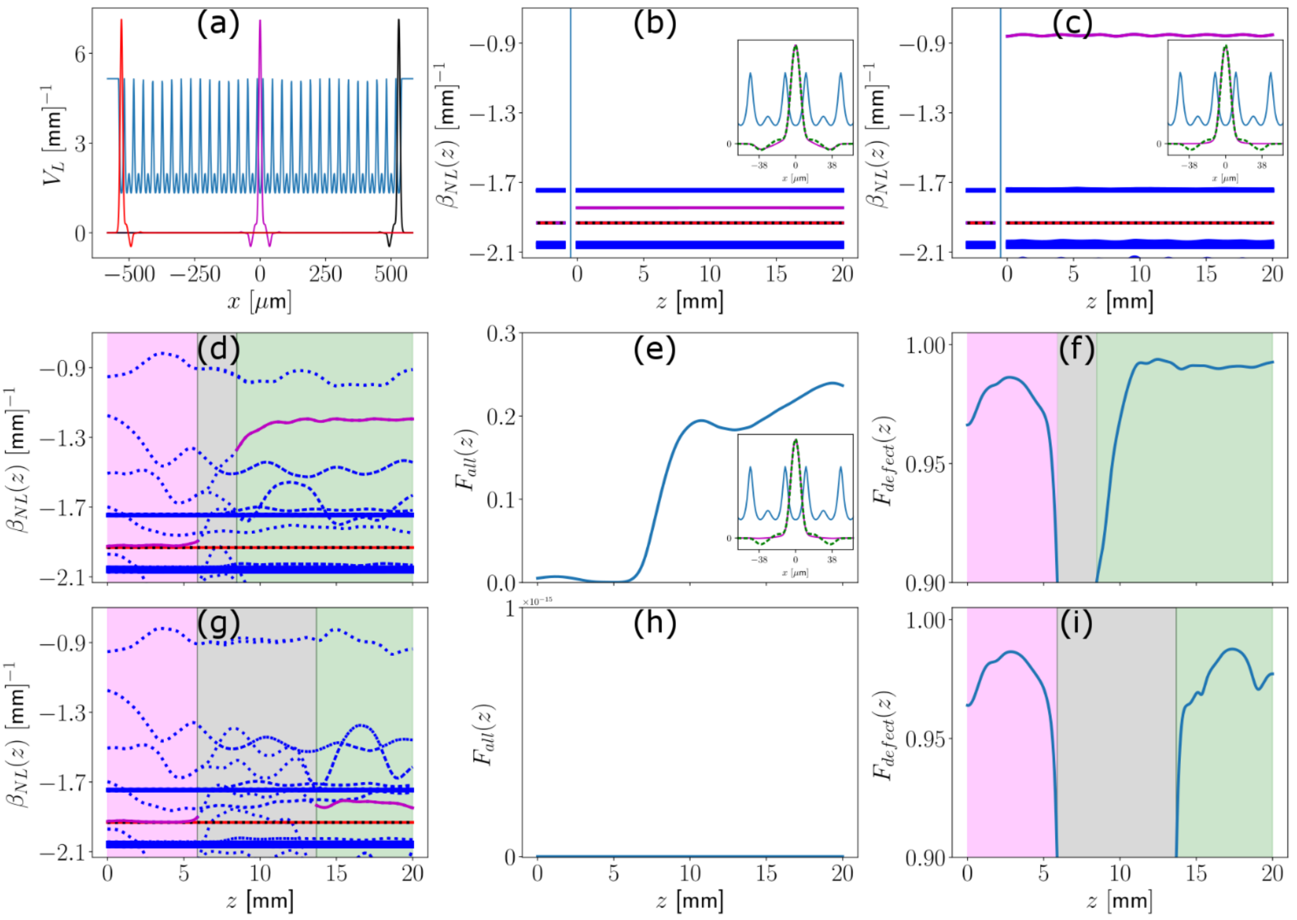

Fig. S1. Dynamics of nonlinear eigenvalues and overlap between linear and nonlinear defect states. (a) The linear SSH lattice (dark blue lines) with one defect state in the center (solid magenta line) and two edge states (solid red and black lines). (b, c) Nonlinear eigenvalues $\beta_{NL,n}(z)$ for $\psi(\mathbf{x}, z = 0) = \sqrt{I_0}\varphi_{L,defect}$ at low (b) and high (c) nonlinearity; for comparison, the linear spectrum $\beta_{L,n}$ is shown for $z < 0$. Magenta line depicts the nonlinear eigenvalue $\beta_{NL,defect}(z)$ of the interface defect mode $\varphi_{NL,defect}$. Red and black lines correspond to the edge modes which are not populated. Thick blue lines are the bands. The insets show the linear topological mode (green dashed line) and nonlinear defect mode (magenta solid line). (d-f) Results for two beams launched in phase towards the defect. (d) Dynamics of the nonlinear eigenvalues $\beta_{NL,n}(z)$; the color notations for the defect mode and the two edge modes are the same as in (b) and (c). Dotted blue lines indicate the nonlinear eigenvalues of modes localized solely due to the nonlinearity. (e) The overlap of the whole beam and the linear defect mode $F_{all}(z)$. (f) The overlap of the nonlinear defect mode and the linear defect mode $F_{defect}(z)$. (g-i) Dynamics for the two beams initially out of phase. The layout is identical to that for (d-f). Three shaded regions (magenta, gray, and green) in (d, f, g, i) correspond to three different stages of nonlinear beam dynamics. See text for details.

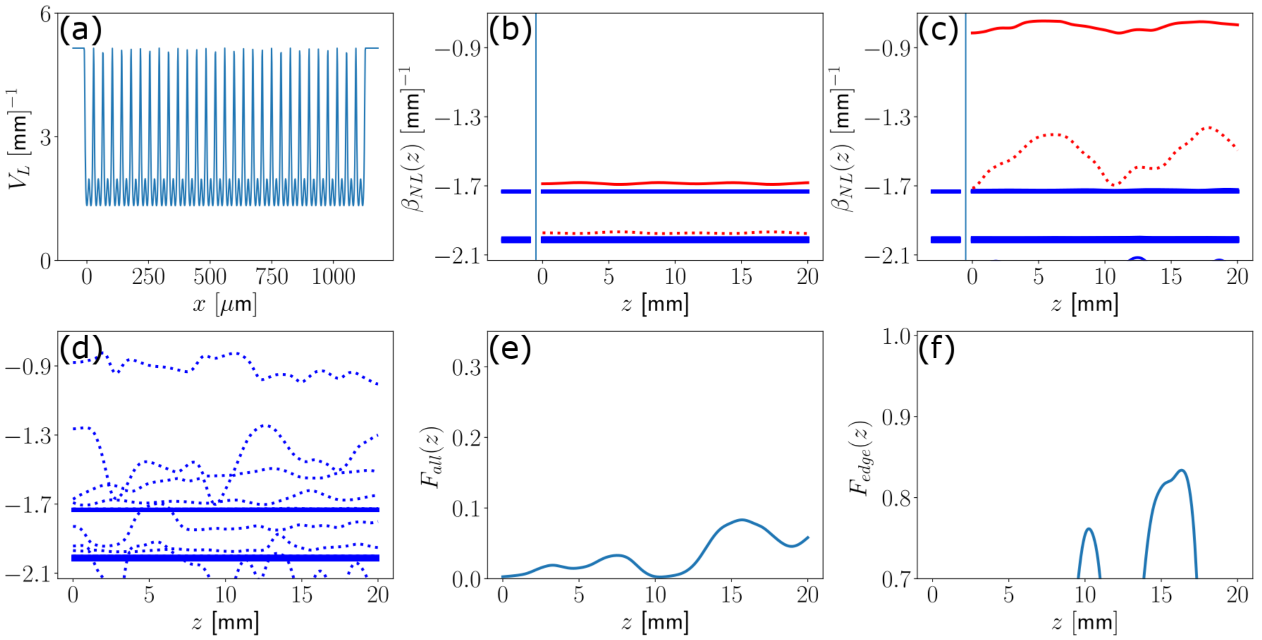

Fig. S2. Dynamics of nonlinear eigenvalues for excitations of SSH lattice in topologically trivial regime. (a) The linear SSH lattice (dark blue lines) in the topologically trivial regime. (b, c) Nonlinear eigenvalues $\beta_{NL,n}(z)$ for $\psi(\mathbf{x}, z=0) = \sqrt{I_0}\varphi_{L,defect}$ at low (b) and high (c) nonlinearity. Red solid and red dotted lines correspond to the nonlinear localized modes. These modes are purely nonlinear and not related to the topology of the SSH lattice. (d-f) Dynamics for a beam launched at an angle towards the edge. (d) Evolution of nonlinear eigenvalues $\beta_{NL,n}(z)$. Thick blue lines correspond to the bands, and dotted blue lines correspond to the (purely) nonlinear localized states. (e) The overlap of the whole beam with the linear edge mode of the topologically nontrivial SSH lattice $F_{all}(z)$; small values indicate that the nonlinearly excited modes are not related to linear topological states. (f) None of the nonlinear localized modes resembles the structure of the linear edge mode of the topologically nontrivial SSH lattice, as seen from $F_{edge}(z)$; occasional and accidental overlaps above 70% arise from the nonlinear dynamics of mode beating, but they do not persist and cannot reach high values of 98-99% presented in Fig. 3 of the main text.
